# Marker of schizophrenia with enduring negative symptoms

**DOI:** 10.1192/j.eurpsy.2022.2001

**Published:** 2022-09-01

**Authors:** E. Dmitrieva, L. Smirnova, A. Semke

**Affiliations:** 1 Tomsk National Research Medical Center of the Russian Academy of Sciences, Mental Health Research Institute, Tomsk, Russian Federation; 2 Mental Health Research Institute, Tomsk National Research Medical Center of the Russian Academy of Sciences, Department Of Endogenous Disorders, Tomsk, Russian Federation

**Keywords:** schizophrenia, proteomics, biomarker, negative symptoms

## Abstract

**Introduction:**

The relevance of this study is determined by the need to search for biological markers of schizophrenia. The detection and validation of such molecules can become the basis for the creation of additional paraclinical diagnostic methods or contribute to the creation of targets for individual pharmacotherapy, which is an important task of modern fundamental medicine.

**Objectives:**

Comparative proteomic analysis of serum in schizophrenic patients with positive and negative symptoms.

**Methods:**

The study includes 10 healthy donors and 27 patients with schizophrenia. Samples preparation included: serum purification from major proteins via affinity chromatography, 1D-PAGE proteins separation, in-gel tryptic hydrolysis, LC-MS/MS mass-spectrometry (Orbitrap Q-exactive HF mass spectrometer, Agilent Technologies). Identification of proteins was carried out using Mascot software Ver. 2.1 («Matrix Science», USA). Proteins for quantitative analysis were selected in view of the DISGENET database. Quantitative LC-MS-SRM analysis of selected protein was performed on QQQ TSQ Vantage (Thermo Scientific) with labeled peptide standards.

**Results:**

Receptor-interacting serine/threonine-protein kinase 1 was selected for quantitative assessment. Significant differences were revealed in the RIPK1 concentrations in the serum of schizophrenic patients with negative and positive symptoms (p=0.02). The serum concentration of RIPK1 in patients with negative symptoms is tenfold in patients with positive symptoms.

**Conclusions:**

Receptor-interacting serine/threonine-protein kinase 1 can be considered a biomarker of negative symptoms of schizophrenia based on a significant increase in serum concentration. *Mass spectrometric analysis was carried out of the “Human Proteome” Core Facility of the Institute of Biomedical Chemistry Moscow. Support by Grant of RSF № 18-15-00053P.*

**Disclosure:**

No significant relationships.

